# *Proteus mirabilis* in Nonpuerperal Breast Infection: A Nosocomial Infection?

**DOI:** 10.1155/S1064744995000652

**Published:** 1995

**Authors:** Ines Kappstein

**Affiliations:** Institute of Environmental Medicine and Hospital Epidemiology University Hospital Freibug Germany


Infectious Diseases in Obstetrics and Gynecology 3:217–218 (1995)
© 1996 Wiley-Liss, Inc.

### *Proteus mirabilis* in Nonpuerperal Breast Infection: A Nosocomial Infection?

I have read with interest the article by Miranda Casas et al.[1] describing the frequent isolation of *Proteus mirabilis* in patients with nonpuerperal breast infection which has not been reported by other authors.[1,2] Working in the field of nosocomial infection control, however, I was surprised to read the almost casual remark of the potential nosocomial origin of this organism ("... thus suggesting that it was a nosocomial infection."). I believe that there are several epidemiologic considerations to be discussed before the conclusion of a (possible) nosocomial origin can be drawn: What were the characteristics of the patients with *P. mirabilis* isolated in mixed or pure culture compared with the other patients? Did the affected women have potential risk factors such as carcinoma of the breast which could predispose them to colonization with "unusual" organisms? Did these women perhaps have a prolonged (or repeated) hospital stay with another organism isolated first before *P. mirabilis* appeared, demonstrating a change in pathogen during hospitalization? Did the pathogens come from an endogenous or an exogenous reservoir? Did the women have prior medical procedures causing transient bacteremia with body flora or, if an exogenous source were likely, what could have been the mode of transmission: contaminated instruments or the hands of staff or anything else? Without considering at least some of these issues, the presumed nosocomial origin of *P. mirabilis* breast infections remains vague and unconvincing.

*Ines Kappstein*
*Institute of Environmental Medicine and Hospital Epidemiology*
*University Hospital*
*Freiburg, Germany*

### REFERENCES

1. Miranda Casas C, Pérez M, Alados JC, et al.: Nonpuerperal breast infection. Infect Dis Obstet Gynecol 3:64–66, 1995.
2. Giamarellou H, Soulis M, Antoniadou A, Gogas J: Periareolar nonpuerperal breast infection: Treatment of 38 cases. Clin Infect Dis 18:73–76, 1994.

Letter to the Editor

Received November 7, 1995
Accepted December 19, 1995

### Author's Reply

We agree with the author's observation that the breast infection by *Proteus mirabilis* that we describe was probably not of nosocomial origin. All patients were healthy women who were not previously hospitalized. As we remarked in our paper, *P. mirabilis* was the only *Enterobacterium* isolated in this infectious syndrome in the absence of a history of surgery or diagnostic traumatic injury. Our phrase, "thus suggesting that it was a nosocomial infection," should therefore read, "thus suggesting that it was not a nosocomial infection."

*C. Miranda Casas*

*M. Pérez*

*J.C. Alados*

*G. Orellana*

*J.M. Aguilar*

*M. de la Rosa*

*Microbiology Service*

*J. Fontes*

*J.A. Miranda*

*Department of Gynecology and Obstetrics*

*Virgen de las Nieves Hospital*

*Granada, Spain*
